# [^11^C]Flumazenil brain uptake is influenced by the blood-brain barrier efflux transporter P-glycoprotein

**DOI:** 10.1186/2191-219X-2-12

**Published:** 2012-03-28

**Authors:** Femke E Froklage, Stina Syvänen, N Harry Hendrikse, Marc C Huisman, Carla FM Molthoff, Yoshihiko Tagawa, Jaap C Reijneveld, Jan J Heimans, Adriaan A Lammertsma, Jonas Eriksson, Elizabeth CM de Lange, Rob A Voskuyl

**Affiliations:** 1 Department of Neurology, VU University Medical Center, De Boelelaan 1117, Amsterdam, 1081 HV, The Netherlands; 2 SEIN - Epilepsy Institute in the Netherlands Foundation (SEIN), Achterweg 5, Heemstede, 2103 SW, The Netherlands; 3 Division of Pharmacology, LACDR, Leiden University, PO Box 9502, Leiden, 2300 RA, The Netherlands; 4 Department of Nuclear Medicine & PET Research, VU University Medical Center, De Boelelaan 1117, Amsterdam, 1081 HV, The Netherlands; 5 Takeda Chemical Industries Ltd, 17-85, Juso-Honmachi 2-Chome, Yodogawa-ku, Osaka, 532-8686, Japan

**Keywords:** Positron emission tomography, PET, tariquidar, rodents, GABA_A _receptors, P-glycoprotein, epilepsy

## Abstract

**Background:**

[^11^C]Flumazenil and positron emission tomography (PET) are used clinically to assess gamma-aminobutyric acid (GABA)-ergic function and to localize epileptic foci prior to resective surgery. Enhanced P-glycoprotein (P-gp) activity has been reported in epilepsy and this may confound interpretation of clinical scans if [^11^C]flumazenil is a P-gp substrate. The purpose of this study was to investigate whether [^11^C]flumazenil is a P-gp substrate.

**Methods:**

[^11^C]Flumazenil PET scans were performed in wild type (WT) (*n *= 9) and *Mdr1a/1b*, (the genes that encode for P-gp) double knockout (dKO) (*n *= 10) mice, and in naive rats (*n *= 10). In parallel to PET scanning, [^11^C]flumazenil plasma concentrations were measured in rats. For 6 of the WT and 6 of the dKO mice a second, [^11^C]flumazenil scan was acquired after administration of the P-gp inhibitor tariquidar. Cerebral [^11^C]flumazenil concentrations in WT and *Mdr1a/1b *dKO mice were compared (genetic disruption model). Furthermore, pre and post P-gp-blocking cerebral [^11^C]flumazenil concentrations were compared in all animals (pharmacological inhibition model).

**Results:**

*Mdr1a/1b *dKO mice had approximately 70% higher [^11^C]flumazenil uptake in the brain than WT mice. After administration of tariquidar, cerebral [^11^C]flumazenil uptake in WT mice increased by about 80% in WT mice, while it remained the same in *Mdr1a/1b *dKO mice. In rats, cerebral [^11^C]flumazenil uptake increased by about 60% after tariquidar administration. Tariquidar had only a small effect on plasma clearance of flumazenil.

**Conclusions:**

The present study showed that [^11^C]flumazenil is a P-gp substrate in rodents. Consequently, altered cerebral [^11^C]flumazenil uptake, as observed in epilepsy, may not reflect solely GABA_A _receptor density changes but also changes in P-gp activity.

## Background

An important potential mechanism of pharmacoresistance in people with epilepsy is inadequate access of antiepileptic drugs to their intrabrain targets. This may be caused by limited drug distribution across the blood-brain barrier (BBB) due to increased activity of multidrug efflux transporters, such as P-glycoprotein (P-gp) [1]. Recent *ex vivo *data suggest that the positron emission tomography (PET) radioligand [^11^C]flumazenil might be a P-gp substrate [2]. Currently, [^11^C]flumazenil is used clinically as a PET tracer for assessment of gamma-aminobutyric acid (GABA)_A _receptor mediated inhibition in epilepsy and to determine focus localization prior to resective surgery [3,4]. If [^11^C]flumazenil is indeed a P-gp substrate, an anticipated increase in P-gp function at the BBB due to epilepsy could lead to a reduction in brain uptake of [^11^C]flumazenil and consequently to erroneous interpretation with respect to GABA-ergic function.

Whereas in humans, the multidrug resistance 1 (*MDR1*) gene encodes for P-gp in mice and rats two genes (*Mdr1a *and *Mdr1b*) encode for two subtypes of P-gp that together perform the same function as human P-gp [5]. To investigate the impact of P-gp on drug distribution, two approaches can be followed. The first is the use of genetically P-gp-disrupted *Mdr1a/Mdr1b *mice (double knockout (dKO), *Mdr1a/1b *dKO) model [6], the second is the pharmacological inhibition of P-gp. A number of compounds have been used for pharmacological inhibition. The third generation P-gp blocker tariquidar is one of the most potent modulators of P-gp [7,8]. It has been shown that P-gp inhibition following intravenous administration of tariquidar in rats occurs fast and has long duration [9,10].

Potentially genetic disruption of the P-gp encoding genes may induce upregulation of other transporters (i.e., multidrug resistance protein 1, and breast cancer resistant protein (Bcrp)1), while chemical inhibition of P-gp function might also inhibit other transporters, such as Bcrp1, in the case of tariquidar [11]. Therefore, in the present study, a combination of both approaches was used to investigate whether [^11^C]flumazenil is indeed a P-gp substrate in rodents, i.e., to assess whether changes in cerebral [^11^C]flumazenil uptake may reflect not only changes in GABA_A _receptor density, but also in P-gp activity. For that reason, [^11^C]flumazenil scans, before and after administration of the P-gp blocker tariquidar (pharmacological inhibition model), were performed in both *Mdr1a/1b *dKO and wild type (WT) mice (genetic disruption model) and in rats.

## Methods

### Animals

*Mdr1a/1b *dKO (FVB.129P2-*Abcb1a^tm1Bor^Abcb1b^tm1Bor ^*N12, male, *n *= 10) and WT (FVB/NTac, male, *n *= 10) mice (Taconic, Ejby, Denmark), and adult Sprague-Dawley rats (Harlan, Horst, The Netherlands, male, *n *= 11) were used. Approval of the institutional animal ethics committee was obtained, and all experiments were carried out in accordance with the Dutch Law on Animal Experimentation and guidelines of the institutional committee on animal experimentation. All animals were kept at a constant temperature of 21°C and a 12-h light/dark cycle, in which the lights were switched on at 8 a.m. Animals had unrestricted access to food (Teklad Global Rodent Diet, Harlan, Madison, WI, USA) and water. Studies were performed after approximately 1 week of habituation.

### Animal preparation and anesthesia

In each mouse a cannula was inserted in the jugular vein and one in the intraperitoneal space 1-2 h prior to PET scanning. Each rat underwent cannulation of the femoral vein and artery 1-2 h before PET scanning. From the start of surgery until the end of scanning, animals were anesthetized with a combination of (S)-ketamine and medetomidine (Domitor, Pfizer, Capelle a/d IJssel, The Netherlands). Mice received an intraperitoneal injection of 75 mg·kg^-1 ^(S)-ketamine (Ketanest S, Pfizer BV, Capelle aan de IJssel, The Netherlands) and 1.0 mg·kg^-1 ^medetomidineMaintenance anesthesia was administered via the intraperitoneal cannula (37.5 mg·kg^-1^·h^-1 ^(S)-ketamine and 0.5 mg·kg^-1^·h^-1 ^medetomidine). Rats first received an intraperitoneal injection of 60 mg·kg^-1 ^ketamine (Ketalar, Parke-Davis, Hoofddorp, The Netherlands) followed by a second intraperitoneal injection of 0.4 mg·kg^-1 ^medetomidine. To maintain anesthesia, an intravenous infusion into the tail vein of 10 mg·kg^-1^·h^-1 ^ketamine and 0.1 mg·kg^-1^·h^-1 ^medetomidine was administered. This was started between 60 and 120 min after the first intraperitoneal injections and, in most animals, the infusion was then continued for the remainder of the experiment.

### Radiochemistry

[^11^C]Flumazenil was synthesized as previously described [12] and 3-10 GBq was formulated in 10 mL of saline containing 7.1 mM NaH_2_PO_4 _and 10% *v/v *ethanol. The specific activity was 178 ± 69 GBq·μmol^-1 ^(mean ± standard deviation (SD)). The radiochemical purity was higher than 99% as assessed by analytical high performance liquid chromatography (HPLC) equipped with radio and ultraviolet detectors. The HPLC analysis did not detect any chemical impurities. The identity of [^11^C]flumazenil was confirmed by comparison of the retention times with the authentic isotopically unmodified (unlabelled) flumazenil.

### PET scanning

Animals were positioned in pairs in a double lutetium oxyorthosilicate/Lu1.8Y0.2SiO5(Ce) or LSO-LYSO layer high resolution research tomograph (Siemens/CTI, Knoxville, TN, USA) PET scanner [13]. First, a transmission scan was acquired using a 740 MBq 2-dimensional fan-collimated ^137^Cs (662 keV) moving point source [14]. Next, a dynamic emission scan was acquired immediately following administration of 7.2 ± 3.3 (mean ± SD) MBq [^11 ^C]flumazenil to each mouse or 24.4 ± 6.7 MBq [^11 ^C]flumazenil to each rat. The injected activity for each of the different animal groups and scans is shown in Table 1. Emission data were acquired for 30 min in 3-dimensional (3D) list mode and rebinned into the following frame sequences: 4 × 30, 3 × 60, 2 × 150, and 4 × 300 s (mice) or 6 × 10, 2 ×30, 3 × 60, 2 × 150, 2 × 300 and 1 × 600 s (rats). Following corrections for decay, dead time, attenuation, randoms and scatter, scans were reconstructed using a 3D ordinary Poisson ordered subsets expectation maximization algorithm for mice and a 3D ordered subsets weighted least squares algorithm for rats, which show comparable quantitative accuracy [14]. This resulted in images with an average spatial resolution of 3 mm full width at half maximum [13].

**Table 1:** Number of mice/rats, together with average (± standard deviation) weight, dose of flumazenil and radioactivity administered

	Mice		Rats			
	
	*Mdr1a/1b*	WT	FMZ	FMZ	FMZ	FMZ
	dKO		dose 4 μg	dose 20	dose 100	dose 400
				μg	μg	μg
Number (*n*)						
Scan 1	10	9	3	3	2	2
Scan 2	6	6	3	3	2	2

Animal weight (g)	31 ± 2	26 ± 2	283 ± 24	267 ± 11	260, 293	265, 270

Radioactivity						
[^11^C]FMZ (MBq)						
Scan 1	7.3 ± 3.0	6.5 ± 2.0	31 ± 8	24 ± 9	30, 34	18, 20
Scan 2	7.0 ± 1.5	7.8 ± 2.0	21 ± 5	18 ± 2	16, 26	21, 16

Exact FMZ dose						
(μg)						
Scan 1	td	td	3.6 ± 0.1	16.3 ± 0.2	83, 87	366, 424
Scan 2	td	td	3.9 ± 0.1	17.9 ± 1.0	85, 89	391, 458

Abbreviations: *dKO *double knockout, *WT *wild type, *FMZ *flumazenil, *td *tracer dose (< 0.1 μg).

Each animal underwent two consecutive [^11^C]flumazenil PET scans separated by 100 min to allow for radioactive decay of carbon-11, with the exception of three *Mdr1a/1b *dKO mice and three WT mice who received only a baseline [^11^C]flumazenil scan. Tariquidar, 15 mg·kg^-1 ^, a dose that completely blocks P-gp function, was administered as a 15-min infusion 35 min before the second scan to all mice and as a 10-min infusion 20 min before the second scan to all rats, providing optimal inhibition conditions of tariquidar [9]. *Mdr1a/1b *dKO mice received tariquidar to ensure that tariquidar does not influence [^11 ^C]flumazenil brain distribution in the absence of functional P-gp and exclude any P-gp-unrelated pharmacokinetic interaction.

In rats the specific activity of [^11^C]flumazenil was modified to study brain distribution of [^11^C]flumazenil at different GABA_A _receptor occupancies; this was done by adding four different doses, 5, 20, 100 or 400 μg, of isotopically unmodified flumazenil to the injection solutions of [^11^C]flumazenil (which after synthesis contained less than 500 ng isotopically unmodified flumazenil). These doses were chosen based on previous studies of receptor occupancy by flumazenil in rats [15]. The exact doses of [^11^C]flumazenil and unmodified flumazenil were calculated based on the specific activity of the tracer solution and the flumazenil concentration in the solution containing the isotopically unmodified flumazenil and are shown in Table 1. Each rat was assigned to one flumazenil dose, i.e., both scans obtained in one rat were performed with the same amount of unlabeled flumazenil. This enabled each rat to act as its own control when treated with tariquidar.

### Flumazenil analysis in rat blood samples

Eight arterial blood samples of 0.1 mL were taken during each scan. Blood samples were diluted immediately using 0.5 mL of 0.42% NaF in water at 0°C to inhibit esterase activity and thus prevent flumazenil metabolism in the samples. All samples were stored at -80°C until analysis.

Flumazenil concentrations in blood were measured using a method published previously, based on HPLC coupled to tandem mass spectrometry (LC-MS/MS) [16]. This analysis was performed in the same laboratory and with the same equipment, i.e., under identical conditions, as described in the study by Liefaard et al [16]. The limit of detection was 0.5 ng·mL^-1 ^. Linear calibration curves were obtained in the range 0.5-1,000 ng·mL^-1 ^.

### Data analysis

PET image data were analyzed using the freely available software package Amide 0.9.2 [17]. Time-activity curves of brain tissue were obtained by drawing a volume of interest (VOI) in three to four adjacent transaxial planes to cover the whole brain of mice. In rats, VOIs were drawn in four adjacent transaxial planes over frontal cortex, a region in the rat brain with high density of GABA_A _receptors. VOI positioning was defined visually using a summation image of the whole scan. Next, VOIs were projected onto all dynamic frames, resulting in brain time-activity curves of [^11 ^C]flumazenil. Time activity curves were normalized for injected dose and body weight, generating standardized uptake value (SUV) curves as a function of time:

$$\text{SUV}\;\text{ = }\;\frac{\text{Average}\;\text{VOI}\;\text{radioactivity}\;\left[\text{Bq}\cdot \text{m}{\text{L}}^{\text{ - 1}}\right]}{\text{Injected dose}\;\left[\text{Bq}\right]\;\text{per body}\;\text{weight}\;\left[\text{g}\right]}$$

For comparison of results, the average SUV over the period from 20-30 min post injection was used. This average is denoted as SUV_brain _and represents the value at pseudoequilibrium between brain and plasma concentrations.

In mice SUV_brain _was compared between *Mdr1a/1b *dKO and WT mice to assess whether [^11^C]flumazenil is a P-gp substrate (genetic disruption model). Furthermore, as a measure of the effect of tariquidar on brain uptake of [^11^C]flumazenil, SUV_brain _was compared in all animals between pre and post tariquidar scans (pharmacological inhibition model). The *Mdr1a/1b *dKO mice served as controls, as they are devoid of P-gp, which is the target of tariquidar. For rats, SUV_brain _was compared for each flumazenil dose group separately to investigate whether P-gp inhibition is affected by receptor occupancy. In addition, in rats, plasma concentrations of flumazenil were compared before and after tariquidar treatment to determine whether tariquidar had an effect on plasma kinetics of flumazenil.

### Statistical analyses

All statistical analyses were performed in SPSS (version 15.0, IBM Corporation, Armonk, USA). The comparison of SUV_brain _between *Mdr1a/1b *dKO and WT mice was carried out by means of independent samples *t *tests with Bonferroni correction to adjust for multiple comparisons. SUV_brain _comparisons before and after P-gp inhibition for each animal group separately (i.e., rats, *Mdr1a/1b *dKO, or WT mice) were performed using paired samples *t *tests, again with Bonferroni correction to adjust for multiple comparisons.

## Results and discussion

Ten *Mdr1a/1b *dKO mice and nine WT mice had successful baseline [^11^C]flumazenil scans. Of all animals, six *Mdr1a/1b *dKO mice, six WT mice and ten rats had two successful [^11^C]flumazenil scans. One WT mouse died during surgery, and one*Mdr1a/1b *dKO mouse and one rat died after tariquidar treatment. The baseline scan of the mouse that died during the second scan has been included in the comparison of *Mdr1a/1b *dKO and WT mice before P-gp blockade, as this animal appeared to be healthy before tariquidar treatment. Clearly, for the comparison before and after tariquidar treatment, both animals that died during the second scan had to be excluded. Table 1 shows the animal characteristics, amount of flumazenil and the activity of [^11^C]flumazenil injected.

### Mice

**Figure 1:**
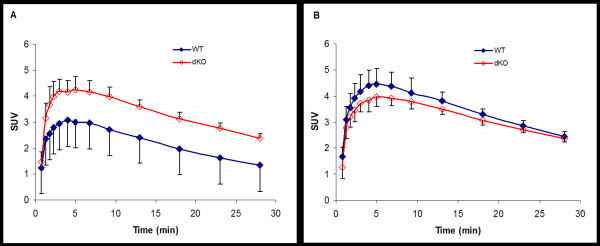
**Average cerebral [^11^C]flumazenil SUV in mice as function of time**. Average cerebral [^11^C]flumazenil SUV in wild type (*closed *symbols) and *Mdr1a/1b *double knockout (*open *symbols) mice before (A) and after (B) i.v. tariquidar administration. *Error bars *represent SD.

**Figure 2:**
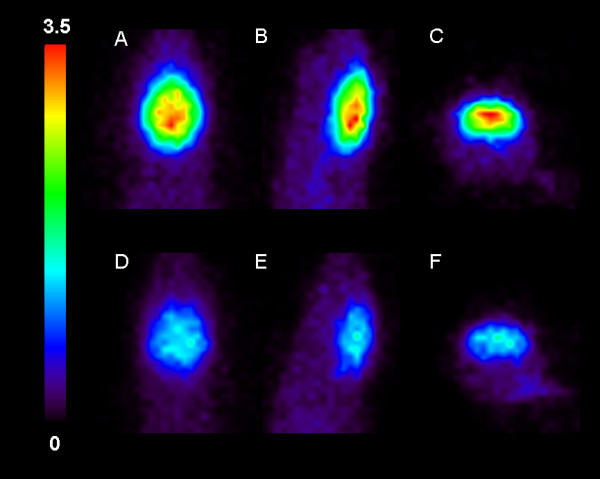
**SUV_brain _images in mice**. Baseline [^11^C]flumazenil SUV_brain _images (20-30 min post injection) for an *Mdr1a/1b *dKO (A-C) and a WT (D-F) mouse. From *left *to *right*: transversal, sagittal and coronal slices.

Before treatment with tariquidar, *Mdr1a/1b *dKO mice showed higher brain uptake than WT mice throughout the entire PET scan (Figure 1). The SUV_brain _of *Mdr1a/1b *dKO mice was 71% higher than the SUV_brain _of WT mice (dKO mice *n *= 10, SUV_brain _= 2.72 ± 0.27 and WT mice *n *= 9, SUV_brain _= 1.59 ± 0.26; *p *< 0.001). Figure 2 shows SUV_brain _images of one *Mdr1a/1b *dKO and one WT mouse, both before tariquidar administration. After P-gp blockade, SUV_brain _increased by 79% in WT mice (*n *= 6, before tariquidar SUV_brain _= 1.48 ± 0.14 and after tariquidar SUV_brain _= 2.66 ± 0.17; *p *< 0.001), while no significant changes were observed in *Mdr1a/1b *dKO mice (*n = *6, before tariquidar SUV_brain _= 2.56 ± 0.20, and after tariquidar SUV_brain _= 2.54 ± 0.11; *p *= 0.8). Furthermore, after tariquidar treatment, the SUV_brain _was not significantly different between WT and *Mdr1a/1b *dKO mice (Figure 1; *p *= 0.2).

### Rats

**Figure 3:**
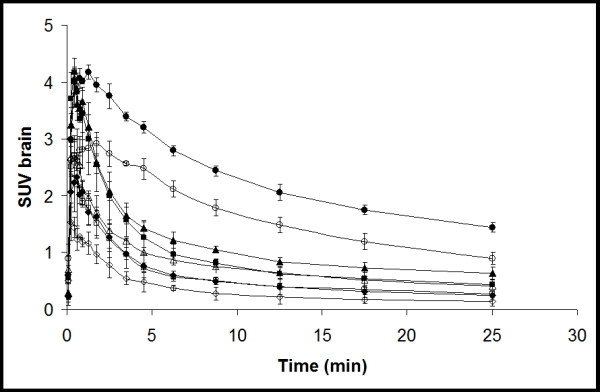
**Average cerebral [^11^C]flumazenil SUV in rats as function of time**. *Circles*, *triangles*, *squares *and *diamonds *indicate the different dose levels of (unlabeled) flumazenil administered, e.g., 4 (*circle*), 20 (*triangle*), 100 (*square*), and 400 μg (*diamond*). *Open *and *closed *symbols indicate data before and after tariquidar treatment, respectively. *Error bars *represent SD.

**Figure 4:**
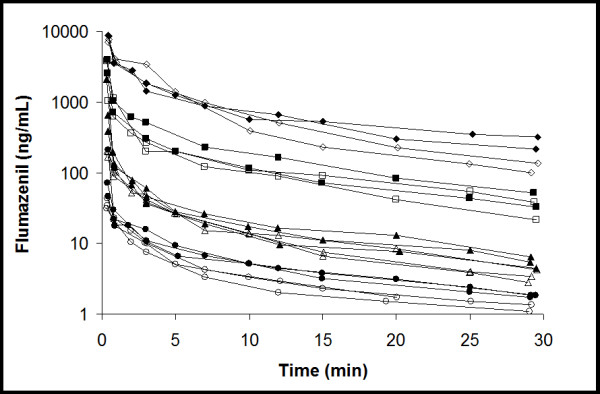
**Average flumazenil plasma concentrations in rats**. *Circles*, *triangles*, *squares *and *diamonds *indicate different dose levels of flumazenil administered, i.e., 4, 20, 100 and 400 μg, respectively. *Open *and *closed *symbols indicate data before and after tariquidar treatment, respectively.

As expected for a receptor ligand, increasing the dose of unlabeled flumazenil resulted in reduced cerebral uptake of [^11^C]flumazenil (Figure 3). SUV_brain _for the group of rats that received 4 μg flumazenil was similar to that of WT mice. The more flumazenil added to the tracer solution, the fewer GABA receptors will be available for binding; as a consequence, the elimination from brain was faster. Treatment with 15 mg·kg^-1 ^tariquidar increased SUV_brain _by 62% (*n *= 10; before P-gp inhibition, SUV_brain _= 0.49 ± 0.30 and after P-gp inhibition, SUV_brain _= 0.79 ± 0.47; *p *= 0.001). This increase in SUV_brain _caused by tariquidar treatment was similar for all flumazenil doses used; 62%, 55%, 59% and 87% for the 4, 20, 100 and 400 μg dose, respectively. Flumazenil concentrations in rat plasma, before and after tariquidar treatment, are shown in Figure 4: there were only small differences between pre and post tariquidar curves toward the end of the scan.

Taken together, absence of P-gp in mice (genetic disruption model) had a similar effect on cerebral [^11^C]flumazenil uptake as inhibition of P-gp (pharmacological inhibition model) in WT mice and rats, showing an approximately 70% higher SUV_brain _both in *Mdr1a/1b *dKO mice and in P-gp inhibited WT rodents compared with WT animals prior to P-gp inhibition. Furthermore, SUV_brain _in *Mdr1a/1b *dKO mice did not change following pharmacological P-gp inhibition. Together, these results demonstrate that [^11 ^C]flumazenil is a P-gp substrate in rodents. Furthermore, [^11^C]flumazenil does not appear to be a Bcrp1 substrate in rodents, as SUV_brain _in *Mdr1a/1b *dKO mice did not change after tariquidar treatment compared to baseline.

Theoretically, it is possible that any increase in cerebral [^11^C]flumazenil concentration was due to an increased plasma [^11^C]flumazenil concentration after tariquidar treatment. To rule out this possibility, plasma flumazenil concentration curves were compared in rats. There was a trend for slightly increased plasma concentrations of flumazenil after tariquidar treatment (Figure 4). The area under the curve in plasma was increased about 30% for the 4, 20 and 100 μg flumazenil doses and 5% for the 400 μg flumazenil dose. The increase in plasma concentrations of flumazenil should actually reduce the tariquidar-caused increase in brain SUV as more isotopically unmodified flumazenil will result in less available receptors for the tracer to bind to and therefore lead a lower SUV. Thus, changes in plasma levels of flumazenil could not account for the observed increase in SUV in the brain but rather reduced the difference between the pre and post tariquidar scans. The somewhat increased plasma concentrations of flumazenil during the second scan was probably a consequence of increased flumazenil distribution to tissues, e.g., the brain, otherwise protected by P-gp, which acted as "depot" compartments from which flumazenil at later time points was redistributed into the plasma. Furthermore, the fact that SUV_brain _in *Mdr1a/1b *dKO mice did not change after pharmacological P-gp inhibition strongly suggests that increased plasma concentration of [^11^C]flumazenil (if any) did not play a significant role. Also, previous test-retest [^11^C]flumazenil studies show no influence of the first scan on the second scan [15].

Previous *in vitro *transport assay studies have suggested that flumazenil is not transported by human P-gp [18,19]. *In vitro *assays might be less sensitive, as several transport assays, such as bidirectional transport and calcein inhibition assays have failed to identify moderate P-gp substrates when these drugs were highly permeable [18]. In line with the findings of this study, an *ex vivo *study [2], described higher [^11^C]flumazenil brain-to-plasma concentration ratios in mice treated with cyclosporine A than in control animals. Cyclosporine A, however, is a first generation P-gp inhibitor, which tends to be less potent and nonselective than tariquidar and may also influence metabolism and elimination of drugs/radioligands via interaction with cytochrome P450 enzymes [20]. Thus, the use of tariquidar is to be preferred over cyclosporine A to adequately study the influence of P-gp alteration on brain distribution of drugs/radioligands. Further, the differences between previous *ex vivo *rodent studies and *in vitro *studies using canine cells (Madin Darby canine kidney) transfected with human MDR1 gene could be due to species differences in BBB transport of [^11^C]flumazenil, which has been observed for other PET radioligands [21]. Although this study showed that higher concentrations of P-gp substrates were found in humans and monkeys compared to rodents, all radioligands that were P-gp substrates in rodents were also in humans as well [21]. In the present *in vivo *study both genetic disruption and pharmacological inhibition of P-gp were used, both providing clear evidence that cerebral uptake of [^11^C]flumazenil can be affected significantly by P-gp activity.

It should be noted that more avid P-gp substrates, such as [^11^C]verapamil, show a much larger (e.g. 1,000%) increase in brain concentrations after the same dose of tariquidar [9,10]. Nevertheless, the main finding of the present study remains valid in that changes in P-gp function could potentially confound interpretation of [^11^C]flumazenil scans, particularly if there are regional differences in P-gp upregulation as indicated by some studies in people with epilepsy [22-24]. Taking the present findings into account, focally decreased [^11^C]flumazenil uptake may not only be due to decreased GABA-ergic function, but may also be the result of increased P-gp activity. If both effects occur simultaneously, the cerebral [^11^C]flumazenil uptake could even be decreased further. Whether this will affect the interpretation of the data depends on the purpose of the study. If the aim is to localize the epileptic focus, decreased [^11^C]flumazenil will, in every case, still help to identify the affected brain region. However, one will not be able to interpret the underlying mechanism(s). Furthermore, if future therapies will be developed with the specific aim either to normalize increased P-gp activity or to compensate for decreased GABA-ergic inhibition, quantitative information is needed on P-gp activity or GABA-ergic function, which cannot be obtained with [^11^C]flumazenil PET scans alone.

PET studies with [^11^C]flumazenil in epileptic rats are needed to clarify whether local changes in cerebral [^11^C]flumazenil uptake in epilepsy are due to decreased GABA-ergic function, P-gp upregulation, or both. Furthermore, a comparison between naive and epileptic rats is needed to assess to which degree P-gp upregulation affects cerebral uptake of [^11^C]flumazenil. Ultimately, [^11^C]flumazenil studies in both pharmacoresistant and pharmacosensitive patients with epilepsy, as well as in healthy volunteers are needed to elucidate this important issue.

## Conclusions

The present study showed that [^11^C]flumazenil is a P-gp substrate (but most likely not a Bcrp1 substrate) in rodents. This finding could have implications for preoperative evaluation of people with epilepsy as altered cerebral [^11^C]flumazenil uptake may be due to changes in both GABA_A _receptor density and P-gp activity, potentially complicating interpretation of data.

## Abbreviations

BBB: Blood-brain barrier; Bcrp: Breast cancer resistant protein; dKO: Double knockout; Mdr: Multidrug resistance gene; PET: Positron emission tomography; P-gp: P-glycoprotein; SUV: Standardized uptake value; VOI: Volume of interest; WT: Wild type.

## Competing interests

The authors declare that they have no competing interests.

## Authors' contributions

Each author contributed significantly to the submitted work. FEF and SS were in charge of the study, acquiring, analyzing, interpreting the data and drafting the manuscript. NHH and CFMM contributed in conception and design of the mice study. MCH was responsible for PET image reconstruction and contributed to data analysis. YT analyzed the rat plasma data. CFMM contributed in to the acquiring of PET data. JCR, JJH, ECML and AAL, with expertise in epilepsy research and preclinical study design and PET, contributed to the design of the study, analysis and interpretation of data. JE was in charge of the radiochemistry. RAV contributed to the design of the study, analysis and interpretation of all data and in drafting the manuscript. All authors read and approved the final manuscript.
